# Continuous electroencephalography (cEEG) in infants with congenital heart disease (CHD)

**DOI:** 10.1038/s41390-023-02520-6

**Published:** 2023-02-15

**Authors:** Swetha Padiyar, Neil Friedman, Elia Pestana-Knight, Ahsan Mossa-Naduvil, Linda Franic, Sarah Worley, Hany Aly

**Affiliations:** 1 Department of Neonatology, Cleveland Clinic Children’s Hospital, Cleveland, OH USA; 2 Department of Neurology, Barrow Neurological Institute at Phoenix Children’s Hospital, Phoenix, AZ USA; 3 Epilepsy Center, Cleveland Clinic, Cleveland, OH USA; 4 Department of Quantitative Health Sciences, Cleveland Clinic, Cleveland, OH USA

## Abstract

**Background:**

Neonates with congenital heart disease (CHD) undergoing cardiopulmonary bypass (CPB) surgery have increased risk of impaired neurodevelopmental outcomes secondary to brain injury. This study aims to characterize pre- and post-operative continuous EEG (cEEG) patterns to detect abnormal cerebral activity in infants with CHD and investigate whether an association exists between the degree of encephalopathy in pre- and post-operative cEEG.

**Methods:**

This retrospective cohort study conducted between 2010 and 2018 at a tertiary hospital in Cleveland, OH included infants with CHD with cEEG monitoring, who underwent CPB surgery within first 6 months of life.

**Results:**

Study included 77 patients, of which 61% were males who were operated at median age 6 days. Pre-operatively, 69% and 87% had normal cEEG and sleep–wake cycles, respectively. Post-operatively, 80% had abnormal cEEG. Longer circulatory arrest time and CPB were associated with lack of continuity (*p* 0.011), excessive discontinuity (*p* 0.007) and prolonged inter-burst interval (IBI) duration (*p* value < 0.001). A significant association existed between severity of encephalopathy in immediate and 24-h post-operative period (*p* value < 0.001).

**Conclusions:**

More than 80% of neonates with CHD have abnormal post-operative EEG. Longer circulatory arrest time and CPB were associated with lack of continuity, excessive discontinuity, and prolonged IBI duration on post-operative EEG.

**Impact:**

- This study shows that majority of neonates with congenital heart disease (CHD) have normal pre-operative EEG with a continuous background and normal sleep–wake cycles. Also, 80% of neonates had abnormal post-operative EEG.
- Longer duration of arrest time and bypass time was associated with lack of continuity, excessive discontinuity, and prolonged IBI duration during post-operative EEG monitoring.
- These findings will help clinicians when counseling parents in the intensive care unit, risk stratification, and long-term neurodevelopmental monitoring in these high-risk patients.

## Introduction

Congenital heart disease (CHD) is one of the most common birth defects in the United States of America (USA), with an incidence of 4/1000 live births to 50/1000 live births as reported by various studies^1^ affecting approximately 40,000 births annually.^1–3^ The incidence of moderate and severe forms of CHD is 6/1000 live births and increases to 19/1000 live births on inclusion of serious bicuspid aortic valve. One-third to one-fourth of these affected newborns require intervention in the form of open-heart surgery in the neonatal period.^2,4^

Neurodevelopmental disability after congenital cardiac surgery is common and is the most consequential sequelae of CHD in neonates undergoing cardiopulmonary bypass (CPB) surgery during infancy. Although timing of brain injury is not precisely known, neonates that undergo open heart surgery in early infancy for CHD are at an increased risk for brain injury and subsequent impaired neurodevelopmental outcomes. As many as 50% of these infants have brain injury documented on pre-operative brain magnetic resonance imaging (MRI).^5–7^ Methods to identify brain injury and predict neurodevelopmental outcomes using non-invasive neurodiagnostic tools such as electroencephalography (EEG) could improve pre-operative and critical post-operative care of these newborns along with long term prognostication.^8^ In addition, guidelines by the American Clinical Neurophysiology Society (ACNS) recommends post-operative EEG monitoring in neonates and infants with CHD that require early surgery.^9^ A study showed that only 55% of such infants had normal pre-operative EEG.^10^

Previous studies evaluating amplitude-integrated EEGs (aEEGs) in neonates with CHD in the pre-operative period have reported abnormal background patterns in 45–63%.^8,11,12^ Studies on aEEGs in neonates with CHD in the intra-operative and post-operative period reported abnormal background pattern in 12–24% patients.^13,14^ Seizure activity in the pre-operative period was infrequent, and subclinical seizures was observed in 15% patients^11^ and clinical seizures in 19% patients.^12^ Prevalence of seizures using aEEG recordings post-operatively was 6–33%.^13–18^ Increased mortality was significantly associated with occurrence of post-operative seizures and delay in recovery of the aEEG background pattern beyond 48 h.^16^

However, there is limited data on continuous EEG (cEEG) to assess neurophysiological background abnormalities in the pre- and post-operative period in infants with CHD undergoing palliative or corrective CPB surgery. The aim of this retrospective consecutive cohort study was to characterize cEEG background patterns, presence of sleep–wake cycle, and seizure activity in the pre- and post-operative period in neonates with CHD undergoing CPB surgery. The secondary aims were (1) to evaluate pre-, intra-, and post-operative factors and its association with cEEG findings prior to and after cardiac surgery and (2) to investigate whether there was an association between pre- and post-operative cEEG patterns and severity of encephalopathy. We hypothesized that infants with CHD have abnormal pre- and post-operative cEEG findings and that these abnormalities are associated with each other.

## Materials and methods

### Study population

This retrospective consecutive cohort study was performed at the neonatal and pediatric intensive care units (NICU and PICU, respectively) of Cleveland Clinic Children’s Hospital, Cleveland, OH, USA. From the medical records database, all infants who had been admitted with a diagnosis of CHD between January 2010 and December 2018 and had been monitored with cEEG were identified. In our hospital, cEEG is a routine procedure in all critically ill infants with CHD prior to and after cardiac surgery. Infants with any type of CHD who underwent CPB surgery in the first 6 months of life were included in the study. Only infants with pre- and post-operative cEEG recordings were included in this study. Neonates with gestational age less than 36 weeks, confirmed genetic disorders or multiple congenital anomalies, and neonates with known underlying neurological disorders were excluded from the study. Infants who underwent cardiac transplantation within 30 months of cardiac surgery were also excluded. The study was approved by the Institutional Review Board (IRB) and Pediatric Institute Research Committee at Cleveland Clinic Foundation.

### Continuous EEG

A 19-channel continuous cEEG recording was performed prior to and for 72 h after cardiac surgery. Post-operative cEEG was started after the patient was transferred to the ICU and hemodynamic stability was achieved. Twenty electrodes were placed according to the international 10–20 montage system (modified for neonates) with collodion adhesive.^9^ The cEEG service includes acquisition and review software, network infrastructure, and trained and licensed EEG technologists and physicians. The Cleveland Clinic EEG laboratory is accredited by ABRET. cEEG was performed using the Nihon–Koden digital video EEG system with a portable EEG acquisition machine networked to the main server allowing EEG review at the bedside and also remotely in the central monitoring unit.

cEEG variables such as background patterns, inter-burst interval (IBI)—amplitude and duration, symmetry, synchrony, presence of sleep–wake cycles, grapho-elements, and epileptic activity were assessed. The cEEGs were classified as normal or abnormal based on background patterns. Normal EEG background was defined as normal continuity and discontinuity (IBI < 4 s in quiet sleep), IBI Amplitude 25–50 µV in awake or active sleep, symmetry and synchrony, spontaneous cycling among wake, active, and quiet sleep and normal grapho-elements. Furthermore, severity of encephalopathy was graded as mild, mild–moderate, moderate, moderate–severe, and severe encephalopathy according to classification of neonatal EEGs by Shellhaas et al. based on EEG background features including the presence or lack of continuity or discontinuity, synchrony, symmetry, IBI amplitude and duration, and grapho-elements seen during most part of the EEG recording.^9,19^ Sleep–wake cycle was graded as normal or absent. Epileptic activity was classified as single seizure, multiple seizures, or status epilepticus. Ictal discharges were characterized for timing, multifocality, lateralization, and anti-seizure medication. Immediate post-operative EEG was defined as bedside cEEG monitoring for initial 60 min in the post-operative period, after hemodynamic stabilization in the intensive care unit (ICU). All neonatal EEGs were interpreted by the pediatric epileptologist team for clinical purposes. For the study, cEEG recordings were assessed independently by two investigators, one a neonatologist (S.P.) with approximately 3 years of experience in cEEG interpretation and second a senior EEG technician (L.F.) with more than 18 years of experience. If any discrepancies in findings were noted, final decisions were made by a pediatric epileptologist. cEEG findings were described for the entire length of available recording for each neonate.

### Clinical parameters

Baseline characteristics that were recorded included demographics, birth history (gestational age, birth weight, Apgar scores, cord pH, mode of delivery), fetal cerebrovascular resistance measured by Doppler’s, type of CHD, and details pertaining to the surgery, including age at surgery, pre- and post-operative neurological abnormality, pre-operative and discharge oxygen saturation, pre-operative and discharge hemoglobin (g/dl), prostaglandin use, The Society of Thoracic Surgeons-European Association for Cardio-Thoracic Surgery (STAT) score, type of surgical repair, time to first chest closure, days till first extubation, extracorporeal support days, CPB time (min), cross-clamp time (min), regional cerebral perfusion time (min), cardiac arrest requiring cardiopulmonary resuscitation, acute kidney injury, intensive care length of stay, total length of stay, use of steroids, and sedatives started in 24–48 h post operatively, were also noted. Neonates were classified as Class 1 (Two ventricles with no aortic arch obstruction), Class 2 (Two ventricles with aortic arch obstruction), Class 3 (One ventricle without arch obstruction), and Class 4 (One ventricle with arch obstruction) based on the American Heart Association (AHA) anatomic classification of CHD.^20^

### Data collection

Data were collected and managed using Research Electronic Data Capture (REDCap), a web-based electronic application through Cleveland Clinic Children’s Hospital.

### Statistical analysis

All statistical analyses were performed using the SAS 9.4 software (SAS Institute, Cary, NC). All analyses were performed on a complete-case basis and all tests were two-tailed and performed at a significance level of 0.05. Data were described using medians and ranges for continuous variables and counts and percentages for categorical variables. Associations of cEEG findings were assessed using non-parametric Kruskal–Wallis tests for categorical variables and Spearman rank correlations for continuous and ordinal findings. *p* values of < 0.05 were considered to indicate statistical significance.

## Results

A total of 98 patients with CHD who underwent surgical correction with CPB were identified. Of these 98 patients, pre- and post-operative cEEG recordings were obtained in 77 patients. Twenty-one patients were excluded from the study. The reasons for not undergoing cEEG monitoring included patients transferred from outside facilities for surgical correction and critical clinical condition in the immediate post-operative period requiring repeat surgical intervention. Among patients included in the study, one patient had missing demographic and surgical details in medical records but had pre- and post-operative cEEG recordings, and this data was used for EEG descriptive purposes and data analysis.

Patient demographic characteristics and neurological assessments are shown in Table 1. Of the 76 patients included, 46 (61%) were males and 72 (95%) belonged to the non-Hispanic ethnic group. The median gestational age was 39 weeks (interquartile range (IQR) 36–42). Surgery in these infants took place at median age of 6 days (IQR 0–180), with median length of ICU stay of 12 days (IQR 1.0–178) and total length of stay 24 days (IQR 3.0–218). Five out of 76 patients died in the post-operative period, with an overall mortality rate of 6.6%. Seventy-two out of 76 neonates (95%) had neurological abnormalities during long-term follow-up. Most common neurological abnormality observed was abnormal tone (75%) followed by developmental delay (62%) and microcephaly (33%). In all, 13% of neonates had pre-operative MRI done, all of which were abnormal showing a stroke in 3 out of 8 neonates. Seventy-five neonates had pre-operative and 35 neonates had post-operative head ultrasound (US) obtained showing abnormal results in 18% of neonates before and after surgery.

**Table 1:** Descriptive statistics and neurological status for infants with congenital heart disease (*N* = 77).

Factor	Total (*N* = 77)	
	*N*	Statistics
Sex, *n* (%)	76	
Male		46 (61)
Female		30 (39)
Age at surgery (days), median (min, max)	76	6.0 (0, 180)
Ethnicity, *n* (%)	76	
Hispanic		4 (5.3)
Non-Hispanic		72 (95)
Gestation (weeks), median (min, max)	76	39 (36, 42)
Head circumference percentile at birth, median (min, max)	76	36 (0.010, 100)
Weight percentile at birth, median (min, max)	76	45 (0.010, 96)
ICU length of stay, median (min, max)	76	12 (1.0, 178)
Total length of stay, median (min, max)	76	24 (3.0, 218)
Deceased, *n* (%)	76	5 (6.6)
Neurological status, *n* (%)	76	
Normal		4 (5.3)
Abnormal		72 (95)
Type of neurological abnormality, *n* (%)	76	
Developmental delay	76	47 (62)
Microcephaly	76	25 (33)
Macrocephaly	76	3 (3.9)
Stroke	76	12 (16)
Motor apraxia/coordination	76	4 (5.3)
Behavioral/emotional	76	4 (5.3)
Seizures	76	12 (16)
ADHD	76	8 (11)
Other	76	57 (75)
Pre-operative brain MRI abnormality, *n* (%) (MRI obtained in 10 patients)	8	
Stroke		3 (38)
Other		5 (63)
Post-operative brain MRI abnormality, *n* (%) (MRI obtained in 6 patients)	5	
Stroke		1 (20)
Other		4 (80)
Pre-operative head US abnormality, *n* (%) (head US obtained in 75 patients)	11	
Stroke		1 (9.1)
Bleed		1 (9.1)
Other		9 (82)
Post-operative head US abnormality, *n* (%) (head US obtained in 35 patients)	9	
Stroke		1 (11)
Other		8 (89)

Statistics are presented as median (min, max) or *N* (column %).

The cardiac defects were Class 1 in 31 neonates (41%), Class 2 in 9 neonates (12%), Class 3 in 11 neonates (14%), and Class 4 in 25 neonates (33%) as shown in Table 2. The median duration of CPB was 118 min (IQR 0–271) and cross-clamp time was 50 min (IQR 0–138). Five neonates (6.6%) were placed on extracorporeal membrane oxygenation (ECMO) with 40% requiring ECMO for 2 days and 40% neonates for 3 days.

**Table 2:** Classification of cardiac defects and surgical procedures (*N* = 77).

Factor	Total (*N* = 77)	
	*N*	Statistics
Class, *n* (%)	76	
Two ventricles with no aortic arch obstruction (Class 1)		31 (41)
Two ventricles with aortic arch obstruction (Class 2)		9 (12)
One ventricle without arch obstruction (Class 3)		11 (14)
One ventricle with arch obstruction (Class 4)		25 (33)
Type of cardiac lesion/s		
Single ventricle physiology (HLHS, DORV, DOLV), *n* (%)		33 (43)
Tetralogy of Fallot, *n* (%)		5 (6.6)
Transposition of great arteries, *n* (%)		25 (33)
AV canal, *n* (%)		6 (7.9)
Heterotaxy, *n* (%)		2 (2.6)
Atrial septal defect, *n* (%)		12 (16)
Ventricular septal defect, *n* (%)		16 (21)
Other, *n* (%)		58 (76)
Type of surgery, *n* (%)		
Single ventricular repair		37 (49)
Biventricular repair		39 (51)
STAT category, *n* (%)	76	
1		7 (9.2)
2		8 (11)
3		14 (18)
4		20 (26)
5		27 (36)
Cross-clamp time (min), median (min, max)	76	50 (0, 138)
Length of time on bypass (min), median (min, max)	76	118 (0, 271)
Days till first chest closure, median (min, max)	76	0 (0, 9.0)
Did patient require ECMO within 7 days of surgery, *n* (%)	76	
No		71 (93)
Yes		5 (6.6)
Days of ECMO, *n* (%)	5	
2		2 (40)
3		2 (40)
4		1 (20)

Statistics are presented as median (min, max) or *N* (column %).

*HLHS* hypoplastic left heart syndrome, *DORV* double outlet right ventricle, *DOLV* double outlet left ventricle, *STAT* The Society of Thoracic Surgeons-European Association for Cardio-Thoracic Surgery, *ECMO* extracorporeal membrane oxygenation.

Pre-operative and immediate and 24-h post-operative cEEG findings are presented in Table 3. Seventy-one neonates (92%) had pre-operative cEEG monitoring, whereas 72 (94%) and 69 (90%) neonates had cEEG monitoring in the immediate and 24-h post-operative periods, respectively. Pre-operatively, 49 neonates (69%) had a normal EEG recording. In all, 87% showed a continuous background pattern with normal sleep wake cycling. Seventeen neonates (24%) had mild encephalopathy; about 7% had moderate-to-severe encephalopathy observed on pre-operative EEG. Post-operatively, 61 neonates (85%) and 57 neonates (83%) had abnormal EEG findings in the immediate and 24-h post-operative period. Immediate post-operative EEG showed discontinuous background activity in 41 neonates (54%) and asynchrony in 33%. Normal sleep–wake cycling was seen only in 20 neonates (28%). Lower median IBI amplitude, 25 microvolts (IQR 5.0–50) and longer IBI duration, 8 s (IQR 2.0–35), were seen in these neonates. During the 24-h post-operative EEG recordings, 57 neonates (83%) had abnormal findings. Discontinuous background activity was observed in 38 neonates (55%) and 22% neonates had asynchrony with minimal improvement in IBI amplitude and duration. Eighteen neonates (25%) had moderate encephalopathy seen on immediate and 24-h post-operative EEG and 4 neonates (6%) had severe encephalopathy. Three out of 77 neonates (3.8%) who underwent CPB surgery developed seizures in the post-operative period; of them, one neonate was observed to have subclinical seizures. We examined the association of duration of cross-clamp time, arrest time and CPB time with EEG background activity (Table 4). Longer duration of cross-clamp time is associated significantly with lack of continuity in EEG background during immediate post-operative (*p* = 0.008) and 24-h post-operative (*p* = 0.03) EEG monitoring. Longer arrest time was associated with lack of continuity (*p* = 0.01) as well as discontinuous (*p* = 0.007) EEG background during 24-h monitoring, whereas prolonged CPB time correlated with longer IBI duration in the 24-h post-operative period.

**Table 3:** Descriptive statistics for EEG findings.

Factor	Pre-operative EEG		Immediate post-operative EEG		24-h post-operative EEG	
	Total (*N* = 77)		Total (*N* = 77)		Total (*N* = 77)	
	*N*	Statistics	*N*	Statistics	*N*	Statistics
EEG obtained, *n* (%)	77		77		77	
No		6 (8)		5 (6)		8 (10)
Yes		71 (92)		72 (94)		69 (90)
EEG results, *n* (%)	71		72		69	
Normal		49 (69)		11 (15)	69	12 (17)
Abnormal		22 (31)		61 (85)	69	57 (83)
EEG continuous, *n* (%)	71	62 (87)	72	34 (47)	69	32 (46)
EEG discontinuous, *n* (%)	71	16 (23)	72	41 (57)	69	38 (55)
EEG symmetry, *n* (%)	71	71 (100)	72	69 (96)	69	66 (96)
EEG synchronous, *n* (%)	71	61 (86)	72	48 (67)	69	53 (78)
Sleep-wake cycle, *n* (%)	71	62 (87)	72	20 (28)	69	23 (33)
Inter-burst interval—amplitude, median (min, max)	71	40 (10, 70)	72	25 (5.0, 50)	69	30 (5.0, 185)
Inter-burst interval—duration, median (min, max)	71	5.0 (2.0, 20)	72	8.0 (2.0, 35)	69	7.0 (3.0, 20)
Severity of EEG, *n* (%)	71		72		69	
Normal		49 (69)		11 (15.3)		12 (17.4)
Mild		17 (24)		28 (38.8)		27 (39.2)
Mild–moderate		1 (1.4)		9 (12.5)		6 (8.7)
Moderate		2 (2.8)		18 (25)		17 (24.6)
Moderate–severe		2 (2.8)		2 (2.8)		3 (4.3)
Severe		0		4 (5.6)		4 (5.8)

Statistics are presented as median (min, max) or *N* (column %).

**Table 4:** Association of cross-clamp time, arrest time, and bypass time with immediate and 24-h post-operative EEG findings.

Factor	Immediate post-op EEG			24-h post-op EEG		
		Statistics	*p* value		Statistics	*p* value
*Cross-clamp time (minutes)*	*N* *=* *69*			*N* = 66		
Post-operative EEG results			0.12			0.067
Normal	11	42.0 [13.0, 51.0]		12	36.0 [13.5, 50.0]	
Abnormal	58	53.5 [32.0, 72.0]		54	53.5 [34.0, 74.0]	
Post-op EEG continuous			***0.008***			***0.037***
No	36	63.0 [41.5, 74.5]		35	65.0 [34.0, 76.0]	
Yes	33	42.0 [14.0, 51.0]		31	42.0 [21.0, 51.0]	
Post-op EEG discontinuous			0.079			0.058
No	30	45.0 [21.0, 52.0]		30	43.0 [21.0, 51.0]	
Yes	39	60.0 [32.0, 74.0]		36	63.0 [33.0, 75.5]	
Post-op EEG symmetry			0.096			0.10
No	3	71.0 [55.0, 138.0]		3	71.0 [55.0, 138.0]	
Yes	66	50.0 [29.0, 67.0]		63	50.0 [29.0, 72.0]	
Post-op EEG synchronous			0.12			0.50
No	24	43.0 [0.00, 71.5]		15	59.0 [34.0, 81.0]	
Yes	45	52.0 [38.0, 67.0]		51	50.5 [30.0, 67.0]	
Post-op EEG sleep-wake cycle			0.31			0.22
No	49	54.0 [34.0, 71.0]		43	55.0 [32.0, 74.0]	
Yes	20	44.0 [22.0, 63.5]		23	46.0 [21.0, 60.0]	
Post-op EEG inter-burst interval—duration	69	0.17 (−0.07, 0.39)	0.17	66	−0.32 [−0.52, −0.08]	***0.009***
Post-op EEG inter-burst interval—amplitude	69	−0.16 (−0.39, 0.08)	0.18	66	0.08 [−0.16, 0.32]	0.52
Severity of post-op EEG	69	0.14 (−0.10, 0.37)	0.24			
*Arrest time (minutes)*	*N* = 65			*N* = 62		
Post-operative EEG results			0.99			0.11
Normal	10	0.00 [0.00, 4.0]		11	4.0 [0.00, 16.0]	
Abnormal	55	0.00 [0.00, 4.0]		51	0.00 [0.00, 0.00]	
Post-op EEG continuous			0.37			***0.011***
No	33	0.00 [0.00, 0.00]		32	0.00 [0.00, 0.00]	
Yes	32	0.00 [0.00, 12.5]		30	0.00 [0.00, 18.0]	
Post-op EEG discontinuous			0.18			***0.007***
No	29	0.00 [0.00, 16.0]		29	0.00 [0.00, 18.0]	
Yes	36	0.00 [0.00, 0.00]		33	0.00 [0.00, 0.00]	
Post-op EEG symmetry			0.30			0.29
No	3	0.00 [0.00, 0.00]		3	0.00 [0.00, 0.00]	
Yes	62	0.00 [0.00, 4.0]		59	0.00 [0.00, 9.0]	
Post-op EEG synchronous			0.11			0.22
No	23	0.00 [0.00, 0.00]		14	0.00 [0.00, 0.00]	
Yes	42	0.00 [0.00, 13.0]		48	0.00 [0.00, 13.0]	
Post-op EEG sleep–wake cycle			0.70			0.48
No	46	0.00 [0.00, 0.00]		40	0.00 [0.00, 0.00]	
Yes	19	0.00 [0.00, 9.0]		22	0.00 [0.00, 9.0]	
Post-op EEG inter-burst interval—duration	65	−0.11 (−0.34, 0.14)	0.40	62	−0.08 [−0.32, 0.17]	0.54
Post-op EEG inter-burst interval—amplitude	65	−0.06 (−0.30, 0.18)	0.62	62	−0.20 [−0.43, 0.05]	0.12
Severity of post-op EEG	65	−0.06 (−0.30, 0.19)	0.66			
*Bypass time (min)*	*N* = 71			*N* = 68		
Post-operative EEG results			0.45			0.073
Normal	11	109.0 [81.0, 134.0]		12	99.0 [69.0, 128.5]	
Abnormal	60	119.5 [86.0, 142.0]		56	124.5 [100.0, 146.0]	
Post-op EEG continuous			0.067			0.062
No	38	123.5 [100.0, 148.0]		37	130.0 [100.0, 155.0]	
Yes	33	115.0 [69.0, 134.0]		31	116.0 [74.0, 134.0]	
Post-op EEG discontinuous			0.45			0.13
No	30	117.0 [81.0, 138.0]		30	117.5 [74.0, 134.0]	
Yes	41	120.0 [88.0, 144.0]		38	127.5 [100.0, 155.0]	
Post-op EEG symmetry			0.25			0.29
No	3	142.0 [100.0, 271.0]		3	142.0 [100.0, 271.0]	
Yes	68	118.5 [82.5, 141.5]		65	120.0 [88.0, 142.0]	
Post-op EEG synchronous			0.27			0.85
No	24	119.5 [73.0, 136.5]		15	127.0 [78.0, 142.0]	
Yes	47	117.0 [99.0, 148.0]		53	120.5 [99.5, 147.0]	
Post-op EEG sleep–wake cycle			0.42			0.070
No	51	120.0 [100.0, 142.0]		45	127.0 [109.0, 144.0]	
Yes	20	117.0 [69.0, 140.0]		23	113.0 [69.0, 134.0]	
Post-op EEG inter-burst interval—duration	71	0.23 (−0.00, 0.44)	0.053	68	−0.47 [−0.64, −0.26]	***<0.001***
Post-op EEG inter-burst interval—amplitude	71	−0.15 (−0.37, 0.09)	0.22	68	0.20 [−0.04, 0.42]	0.11
Severity of post-op EEG	71	0.15 (−0.08, 0.37)	0.20			

Statistically significant *p* values are bold and italicized.

Statistics are presented as median [25th, 75th percentiles] with Kruskal–Wallis test or Spearman’s correlation (95% CI).

Association between the severity of EEG findings and encephalopathy in the pre-operative, immediate post-operative, and 24-h post-operative period is shown in Tables 5 and 6. Although, EEG severity classification in the pre-operative period showed no association with immediate post-operative EEG, there was a significant association between EEG severity classification in pre-operative period with 24-h post-operative EEG severity classification (*p* = 0.002). A significant association was also observed between EEG severity classification in the immediate and 24-h post-operative period (*p* < 0.001).

**Table 5:** Associations between pre- and post-operative EEG severity.

Factor	Pre-op EEG severity										Spearman correlation (95% CI)	*p* value
	Normal		Mild		Mild–moderate		Moderate		Moderate–severe			
	*N*	*N* (%)	*N*	*N* (%)	*N*	*N* (%)	*N*	*N* (%)	*N*	*N* (%)		
Severity of immediate post-op EEG, *n* (%)	44		18		1		2		2		0.10 (−0.15, 0.35)	0.42
Normal		7 (16)		3 (17)		0 (0)		1 (50)		0 (0)		
Mild		20 (45)		7 (39)		0 (0)		0 (0)		1 (50)		
Mild–moderate		6 (14)		3 (17)		0 (0)		0 (0)		0 (0)		
Moderate		9 (20)		3 (17)		1 (100)		0 (0)		1 (50)		
Moderate–severe		1 (2.3)		1 (5.6)		0 (0)		0 (0)		0 (0)		
Severe		1 (2.3)		1 (5.6)		0 (0)		1 (50)		0 (0)		
Severity of post-op 24-h EEG, *n* (%)	40		18		1		2		2		0.38 (0.14, 0.62)	***0.002***
Normal		9 (23)		2 (11)		0 (0)		0 (0)		0 (0)		
Mild		18 (45)		6 (33)		0 (0)		0 (0)		1 (50)		
Mild–moderate		5 (13)		1 (5.6)		0 (0)		0 (0)		0 (0)		
Moderate		8 (20)		5 (28)		1 (100)		1 (50)		1 (50)		
Moderate–severe		0 (0)		3 (17)		0 (0)		0 (0)		0 (0)		
Severe		0 (0)		1 (5.6)		0 (0)		1 (50)		0 (0)		

Statistically significant *p* values are bold and italicized.

*CI* confidence interval.

*p* value from Spearman correlation.

**Table 6:** Associations between immediate post-operative and 24-h post-operative EEG severity.

Factor	Immediate post-op EEG severity												Spearman correlation (95% CI)	*p* value^1^
	Normal		Mild		Mild–moderate		Moderate		Moderate–severe		Severe			
	*N*	*N* (%)	*N*	*N* (%)	*N*	*N* (%)	*N*	*N* (%)	*N*	*N* (%)	*N*	*N* (%)		
Severity of post-op 24-h EEG, *n* (%)	10		26		9		17		2		4		0.56 (0.35, 0.76)	***<0.001***
Normal		7 (70)		3 (12)		0 (0)		2 (12)		0 (0)		0 (0)		
Mild		0 (0)		17 (65)		6 (67)		3 (18)		0 (0)		0 (0)		
Mild–moderate		0 (0)		4 (15)		1 (11)		0 (0)		0 (0)		1 (25)		
Moderate		2 (20)		2 (7.7)		2 (22)		10 (59)		1 (50)		0 (0)		
Moderate–severe		1 (10.0)		0 (0)		0 (0)		1 (5.9)		1 (50)		0 (0)		
Severe		0 (0)		0 (0)		0 (0)		1 (5.9)		0 (0)		3 (75)		

Statistically significant *p* values are bold and italicized.

*CI* confidence interval.

*p* value from Spearman correlation.

## Discussion

To the best of our knowledge, this is the first study to report characteristics of cEEG findings including EEG background findings and its associations in the pre- and post-operative periods in infants with CHD who undergo surgical correction in the first 6 months of life. This study showed that (1) majority of infants with CHD had a normal EEG pre-operatively with continuous background activity; (2) longer duration of arrest time and CPB was associated with lack of continuity, excessive discontinuity, and prolonged IBI duration during post-operative EEG monitoring; and (3) a significant association was noted between severity scale of pre-operative and 24-h post-operative EEG findings

Our results demonstrated a high rate (95%) of neurological abnormalities observed among these neonates during subsequent outpatient clinical follow-up. Most common neurological abnormality in our study population was abnormal tone in 75% neonates followed by developmental delay (62%) and microcephaly (33%). A previous study of 2 forms of CPB for correction of transposition of the great arteries (TGA) noted a neurological abnormality in up to 37% of the patients.^21–24^ Another study demonstrated an incidence of major neurological disabilities in survivors with hypoplastic left heart syndrome exceeds 60%.^25,26^ Recent studies have recognized that more than half of newborns with complex CHD have clinical evidence of neurological abnormalities on examination before surgery and these are a significant risk factor for later neurodevelopmental impairment.^24,27,28^ Dittrich et al. found that 27% of patients in CHD group had significant developmental delay and 30% of them had abnormalities on neurological exam.^29^ Limperopoulous et al. also demonstrated similar rates of impairment and over 40% with abnormal neurological exam that persisted through school age.^30^

In our study, pre-operative brain MRI was performed in 13% neonates, and 8 out of 10 of these neonates (80%) had abnormal MRI result showing white matter injury/periventricular leukomalacia or stroke. These results are consistent with data reported in previous studies.^6,31–34^ Mahle et al. demonstrated that, in patients with CHD, 25% had PVL or infarction on pre-operative MRI.^34^ In another study by Licht et al., 53% had developmental and/or acquired brain lesions on pre-operative MRI.^33^ Pre- and post-operative cranial US were abnormal in 15–18% neonates, respectively (Table 1). Some studies in full-term neonates have found an abnormal cranial US in 42–59% of cases before surgery.^35,36^

In our study population, median cross-clamp time and CPB time was 50 and 118 min, respectively (Table 2). Prolonged cross-clamp time was associated with lack of continuity in EEG background during the immediate post-operative period (*p* = 0.008) and lack of continuity and longer IBI duration in the 24-h post-operative period (*p* = 0.009). Longer duration of arrest time (deep hypothermic circulatory arrest (DHCA)) and CPB time was associated with lack of continuity (*p* = 0.011) and excessive discontinuity (*p* = 0.007) and prolonged IBI duration (*p* < 0.001) (Table 4), both consistent with abnormal EEG background pattern. Initial studies of acquired brain injury focused on the operative period and CPB technique. Prolonged circulatory arrest time is a major risk factor for neurodevelopmental impairments.^21,22,24,37^ Hence, neonates undergoing cardiac surgery requiring prolonged CPB or DHCA showing lack of continuity, excessive discontinuity, and prolonged IBI duration during immediate and 24-h post-operative EEG monitoring should be considered at high risk for poor neurological outcomes and close long-term follow-up should be pursued in these infants.^38–41^

EEG background activity has been recognized as an important measure of functional brain maturation and impairment.^42^ In our cohort, 69% of neonates had normal EEG findings pre-operatively, with 87% showing continuous background activity and the presence of sleep–wake cycles. About 7% had moderate-to-severe encephalopathy pre-operatively. More than two-thirds of neonates who underwent cardiac surgery had an abnormal EEG tracing during the immediate and 24-h post-operative period and more than 50% showed excessive discontinuity with sleep–wake cycles seen in only one-third of these patients (Table 3). This finding is in line with findings of normal aEEG background reported by Toet et al.^43^ in infants undergoing open-heart surgery for TGA and a study conducted by Gunn et al. with 77% infants showing normal background pattern prior to surgery.^16^ Sleep–wake cycling was present in 97% of the neonates with normal cerebral function prior to surgery.^11,13^ Majority of neonates in the post-operative period had mild encephalopathy (39%) and one-fourth of them had moderate encephalopathy. In comparison to previous aEEG studies by Latal et al.,^13^ which showed discontinuous background in 22% neonates with CHD, our study noted a higher occurrence of excessive discontinuity in EEG background post-operatively. Another study showed that neonates with brain injury spent significant time in discontinuous EEG patterns (trace alternant) and trace discontinue.^44^ Abnormal EEG background patterns could be influenced by treatment with sedatives and pain medication as stated in prior studies.^11,45,46^ Prevalence of seizures was lower in our study population at 3.8%. Previous studies have reported 11–19% prevalence of post-operative seizures using cEEG or aEEG.^15–18^ Our study sheds some light on the association of severity of pre-operative EEG classification compared to severity of immediate and 24-h post-operative EEG classification. There was a significant association between severity of encephalopathy in the pre-operative period with 24-h post-operative EEG severity. A significant associationwas observed between severity of encephalopathy detected on cEEG in the immediate and 24-h post-operative period. Hence, neonates with higher grades of encephalopathy prior to surgery are at a risk of having severe encephalopathy in the post-operative period.

This study has strengths and limitations. A major strength is that this study used continuous conventional 10–20 system EEG for neuromonitoring of neonates with CHD. cEEG has higher sensitivity and specificity to detect neonatal seizures compared to aEEG. Secondly, the study evaluated various components of EEG findings and used this data for objective classification of EEG background based on severity. However, this study has limitations as well. First, not all neonates were monitored for a full 72 h post-operatively. Only a small number of neonates included in the study had brain MRI obtained in pre- and post-operative period. This cohort study included a wide spectrum of CHD types that might have different types of cEEG abnormalities further preventing subgroup analysis. Another limitation is that neonates included in the study did not have EEG monitoring intra-operatively. Future studies performing intraoperative cEEG to identify real-time changes in EEG findings in respect to cardiac interventions are needed. Long-term neurodevelopmental data were collected for the same cohort and will be reported as subsequent results in a study conducted by the same authors.

## Conclusion

In summary, majority of neonates with CHD have normal pre-operative cEEG with a continuous background and presence of sleep–wake cycles. In all, 80% of neonates with all classifications of CHD had abnormal post-operative EEG with excessive discontinuity, lack of sleep–wake cycling, and mild-to-moderate encephalopathy. Longer duration of circulatory arrest time and CPB was associated with lack of continuity, excessive discontinuity, and prolonged IBI duration during post-operative EEG monitoring. It is interesting to report a significant association between severity classification of pre-operative and 24-h post-operative EEG findings indicating that timing and mechanisms of brain injury in neonates with CHD is multifactorial. It is important to consider these findings to facilitate counseling parents in the ICU, risk stratification, and long-term neurodevelopmental monitoring in high-risk patients. Further studies are needed to assess the relationship between cEEG background findings and neurodevelopmental outcomes.

## Data Availability

The datasets generated during and/or analyzed during the current study are available from the corresponding author on reasonable request.
