# Correction: Continuous electroencephalography (cEEG) in infants with congenital heart disease (CHD)

**DOI:** 10.1038/s41390-025-04362-w

**Published:** 2025-08-27

**Authors:** Swetha Padiyar, Neil Friedman, Elia Pestana-Knight, Ahsan Mossa-Naduvil, Linda Franic, Sarah Worley, Hany Aly

**Affiliations:** 1 Department of Neonatology, Cleveland Clinic Children’s Hospital, Cleveland, OH USA; 2 Department of Neurology, Barrow Neurological Institute at Phoenix Children’s Hospital, Phoenix, AZ USA; 3 Epilepsy Center, Cleveland Clinic, Cleveland, OH USA; 4 Department of Quantitative Health Sciences, Cleveland Clinic, Cleveland, OH USA

Correction to: *Pediatric Research* 10.1038/s41390-023-02520-6, published online 15 February 2023

The article “Continuous electroencephalography (cEEG) in infants with congenital heart disease (CHD)”, written by Swetha Padiyar, Neil Friedman, Elia Pestana-Knight, Ahsan Mossa-Naduvil, Linda Franic, Sarah Worley and Hany Aly, was originally published under exclusive licence to the International Pediatric Research Foundation, Inc 2023. As a result of the subsequent decision to publish the article under the open access model, the article’s copyright notice was changed on 23 July 2025 to © The Author(s) 2025 and the article is now distributed under a Creative Commons Attribution 4.0 International License, which permits use, sharing, adaptation, distribution and reproduction in any medium or format, as long as you give appropriate credit to the original author(s) and the source, provide a link to the Creative Commons licence, and indicate if changes were made.

The images or other third party material in this article are included in the article’s Creative Commons licence, unless indicated otherwise in a credit line to the material. If material is not included in the article’s Creative Commons licence and your intended use is not permitted by statutory regulation or exceeds the permitted use, you will need to obtain permission directly from the copyright holder.

To view a copy of this licence, visit http://creativecommons.org/licenses/by/4.0.

